# Os Escores HEART, TIMI e GRACE para Predição de Eventos Cardiovasculares Adversos Maiores no Período de 30 Dias na Era de Troponina I de Alta Sensibilidade

**DOI:** 10.36660/abc.20190206

**Published:** 2020-05-22

**Authors:** Felipe Torralba, Alberto Navarro, Juan Castellanos-de la Hoz, Carlos Ortiz, Alberth Botero, Freddy Alarcón, Nicolas Isaza, Daniel Isaza

**Affiliations:** 1 Fundacion Cardioinfantil Instituto de Cardiologia Bogota Cundinamarca Colômbia Fundacion Cardioinfantil Instituto de Cardiologia, Bogota Cundinamarca - Colômbia

**Keywords:** Doenças Cardiovasculares/mortalidade, Dor no Peito, Infarto do Miocárdio, Predição, Medição de Risco, Fatores de Risco, Troponina, Isquemia Miocárdica

## Abstract

**Fundamento:**

Múltiplos sistemas de pontuação têm sido elaborados para calcular o risco de eventos cardiovasculares adversos maiores (MACE) em pacientes com dor no peito. Não há dados que avaliem se o escore HEART tem um desempenho superior a TIMI e GRACE para a predição de MACE, especialmente na era de troponina I de alta sensibilidade e em uma população exclusivamente latino-americana.

**Objetivo:**

Comparar o desempenho dos escores HEART, TIMI e GRACE para a predição de MACE em 30 dias de acompanhamento, em pacientes atendidos com dor no peito no departamento de emergência.

**Métodos:**

Os escores HEART, TIMI e GRACE foram analisados em 519 pacientes com dor no peito no departamento de emergência. O desfecho primário foi a ocorrência de MACE no período de 30 dias. O desempenho do escore HEART foi comparado com o dos escores TIMI e GRACE utilizando o teste de DeLong, considerando estatisticamente significativos os valores de p de 0,05.

**Resultados:**

Um total de 224 pacientes (43%) apresentaram MACE no período de 30 dias. A estatística C para os escores HEART, TIMI e GRACE foi de 0,937, 0,844 e 0,797 respectivamente (p < 0,0001). Uma pontuação de 3 ou menos no escore HEART apresentou uma sensibilidade de 99,5% e um valor preditivo negativo de 99% para classificar pacientes de baixo risco de maneira correta; ambos os valores foram mais elevados do que aqueles obtidos pelos outros escores.

**Conclusão:**

O escore HEART, em um período de 30 dias, prediz eventos cardiovasculares, mais eficazmente, em comparação com os outros escores. Troponinas de alta sensibilidade mantêm a superioridade previamente demonstrada deste escore. Este escore oferece uma identificação mais precisa dos pacientes de baixo risco. (Arq Bras Cardiol. 2020; [online].ahead print, PP.0-0)

## Introdução

A dor no peito é uma das queixas mais comuns em pacientes que são atendidos no departamento de emergência, com aproximadamente 15 milhões de consultas nos Estados Unidos e na Europa.^1^ Estima-se que 55% destes pacientes tenham uma causa não cardíaca para a dor no peito e que apenas um quinto seja definitivamente diagnosticado com síndromes coronárias agudas.^1 , 2^ Aproximadamente 85% dos pacientes com dor no peito são internados, embora até 60% dos casos pudessem ser tratados no ambulatório.^3^

Na Colômbia, as doenças cardiovasculares também são uma causa de alta mortalidade; entre essas, a cardiopatia isquêmica foi a causa principal durante a década anterior, correspondendo a 49,5% do total desse grupo.^4 , 5^ Os custos anuais do tratamento de pacientes com dor no peito de causas não cardíacas podem chegar até 8 bilhões de dólares nos EUA e a aproximadamente 3,9 bilhões de dólares na Colômbia.^6^ Estas despesas originam-se principalmente dos custos-leito diários e dos estudos radiológicos e laboratoriais.^2 , 7 , 8^ Este impacto econômico significativo tem incentivado esforços para desenvolver alternativas que possibilitem o uso mais eficiente de recursos, sobretudo em países com orçamentos de saúde limitados.^3 , 8 , 9^

O desenvolvimento de uma ferramenta para determinar, com precisão, o risco de eventos cardiovasculares adversos maiores (MACE) nestes pacientes é essencial, e sistemas de pontuação como o TIMI e o GRACE foram criados para enfrentar este problema.^10 , 11^ Mais recentemente, o escore HEART foi criado, sendo o primeiro que foi projetado de maneira prospectiva para predizer MACE.^12 - 14^

O escore HEART superou os escores TIMI e GRACE em populações asiáticas, europeias e norte-americanas.^11 , 15^ O presente estudo visou comparar a precisão destes escores para predizer MACE em um grupo de pacientes latino-americanos com dor no peito que foram atendidos em um centro de referência cardiovascular. Até onde sabemos, este é o primeiro estudo prospectivo desse tipo.

## Métodos

Trata-se de um estudo prospectivo observacional de testes diagnósticos realizados na Fundación Cardioinfantil, localizada em Bogotá, Colômbia. É um hospital de alta complexidade, especializado em medicina cardiovascular, com uma média mensal de 9.000 consultas de emergência, 15% das quais correspondem a emergências cardiovasculares.

Foram incluídos no estudo os pacientes com idade superior a 18 anos que foram atendidos no departamento de emergência com dor aguda no peito entre agosto de 2017 e fevereiro de 2018. De acordo com o protocolo institucional, os pacientes foram avaliados pelo cardiologista; foi realizada a eletrocardiografia, e foi medida a troponina I de alta sensibilidade (TnI-as) inicialmente e 3 após horas, caso necessário, utilizando ARCHITECT STAT assay (Abbot, Lake Bluff, IL, EUA).

Foi diagnosticado infarto agudo do miocárdio (IAM) quando os valores de TnI-as foram superiores a 0,026 ng/mL (valor de referência 0,0 – 0,026 ng/ml). Neste caso, os pacientes foram internados para atendimento hospitalar, arteriografia coronariana e revascularização percutânea ou cirúrgica. Quando os valores foram negativos, mas a dor foi considerada de probabilidade intermediária ou alta, os pacientes foram internados para avaliação subsequente com uma estratégia de estratificação não invasiva.

Foram excluídos do estudo os pacientes com infarto do miocárdio com elevação do segmento ST e causas não cardíacas de dor no peito, tais como pneumonia, trauma ou dor psicogênica.

Foram calculados os escores HEART, TIMI e GRACE para este grupo de pacientes no momento da consulta.

### Escores de risco

Os métodos para calcular os escores GRACE, TIMI e HEART foram descritos em artigos prévios e estarão resumidos em breve.^14 , 16 , 17^ Foram realizados os cálculos dos escores utilizando as informações dispostas no registro médico eletrônico, o primeiro electrocardiograma no momento de apresentação e os primeiros valores laboratoriais medidos, incluindo a medição da troponina com a dosagem de TnI-as.

O escore HEART consiste nas seguintes 5 variáveis categóricas: histórico médico do paciente, eletrocardiograma, idade, fatores de risco para doença cardíaca coronária e troponina. Cada variável possui um valor máximo de 2 pontos, correspondendo a uma pontuação máxima de 10, a qual indica um paciente com risco máximo. O escore GRACE consiste nas seguintes 5 variáveis categóricas: idade, frequência cardíaca, pressão arterial, creatinina e classe Killip; e as seguintes 3 variáveis nominais: parada cardíaca, desvio do segmento ST e elevação da troponina. Cada item corresponde a um valor, e a soma desses valores determina o risco de MACE. Finalmente, o escore TIMI consiste nas seguintes 7 variáveis nominais dicotômicas: idade acima de 65 anos, mais de 3 fatores de risco para doença arterial coronariana, estenose arterial coronariana significativa, sintomas de angina grave, desvio do segmento ST, uso de aspirina durante a última semana e elevação da troponina. A pontuação máxima do TIMI é 7, pontuações mais altas indicando riscos mais altos.

### Ética

O estudo foi realizado de acordo com os princípios estabelecidos na Declaração de Helsinki da Associação Médica Mundial, o Código de Nuremberg e as Diretrizes Internacionais para pesquisa envolvendo seres humanos da Organização Mundial da Saúde, bem como com as regulações nacionais relacionados a cuidados básicos em saúde. O estudo foi aprovado pelo comitê de ética e pesquisa da Fundación Cardioinfantil e os pacientes incluídos neste estudo forneceram o seu consentimento informado esclarecido.

### Gerenciamento dos dados

Os dados incluíram as informações demográficas dos pacientes e as informações sobre histórico clínico, valores laboratoriais, achados eletrocardiográficos e sinais vitais.

Os valores laboratoriais incluíram creatinina e TnI-as, sendo os exames atualmente aplicados pelo protocolo institucional.

O cardiologista assistente avaliou o eletrocardiograma de 12 derivações de acordo com as diretrizes da American Heart Association.^18^ Quando necessário, o exame foi submetido a um segundo cardiologista cego para avaliação.

Foi criado um banco de dados criptografado ao qual somente os autores do estudo tiveram acesso, e foi desenvolvido um algoritmo para o cálculo automático dos escores de risco.

### Acompanhamento

O acompanhamento foi realizado no 30º dia, revisando-se o registro médico eletrônico e empregando uma pesquisa telefônica. Foi aplicado um formato estruturado com 4 perguntas claras sobre a ocorrência de eventos cardiovasculares importantes (morte, infarto do miocárdio, revascularização cirúrgica ou revascularização percutânea), para determinar a presença do desfecho primário.

### Desfechos

Foi diagnosticado IAM quando os valores de troponina foram acima do percentil 99 dos valores de referência (TnI-as > 0,026 ng/mL) e evidência de isquemia miocárdica foi documentada no eletrocardiograma. Foram aplicados os critérios descritos pela terceira definição universal de IAM, a qual era válida no momento da elaboração do protocolo.^19^ Também foram definidos o infarto do miocárdio com elevação do segmento ST (IMCST), o infarto do miocárdio sem elevação do segmento ST (IMSST) e sinais de isquemia de acordo com as diretrizes validadas no momento da elaboração do protocolo.^20^

A revascularização percutânea foi definida como qualquer intervenção por meio de um cateter nas artérias coronárias e a revascularização cirúrgica foi definida como qualquer cirurgia cardíaca na qual enxertos de artéria coronária foram realizados. MACE foram definidos como morte por qualquer causa, infarto do miocárdio e cirurgia ou revascularização percutânea. Foi concluído o acompanhamento 30 dias após a internação no departamento de emergência.

### Análise estatística

Nós calculamos uma amostra de 550 pacientes, para obter 185 MACE utilizando o método de sensibilidade máxima de Simel e Samsa,^21^ com a finalidade de ter uma potência de 80% e um intervalo de confiança de 95% com um erro alfa de 5%. Para cada escore, o melhor valor de corte foi calculado utilizando a índice de Youden,^22^ considerando significativos os valores de p de 0,05. Subsequentemente, foram calculadas a estatística C, a razão de verossimilhança (RV) positiva e negativa, a sensibilidade e a especificidade. Em seguida, a RV foi calculada para cada estrato de risco. Foi calculada a diferença entre as RV utilizando o teste para proporções binomiais adequadas (o teste de qui-quadrado e o teste exato de Fisher), considerando significativos os valores de p de 0,05.

A área sob a curva para cada teste foi calculada e comparada utilizando o teste não paramétrico de DeLong (p = 0,05). Por fim, também foi realizado um teste de calibração para cada escore para comparar os MACE esperados e reais na população estudada, de acordo com o método do cinto de calibração descrito por Finazzi S, et al.,^23^ do Italian Group for the Evaluation of Interventions in Intensive Care Medicine (GiViTi).^23^

A análise foi realizada utilizando-se o programa estatístico R, versão 3.3.3 (the R Foundation for Statistical Computing, Viena, Áustria).

## Resultados

**Figura 1:**
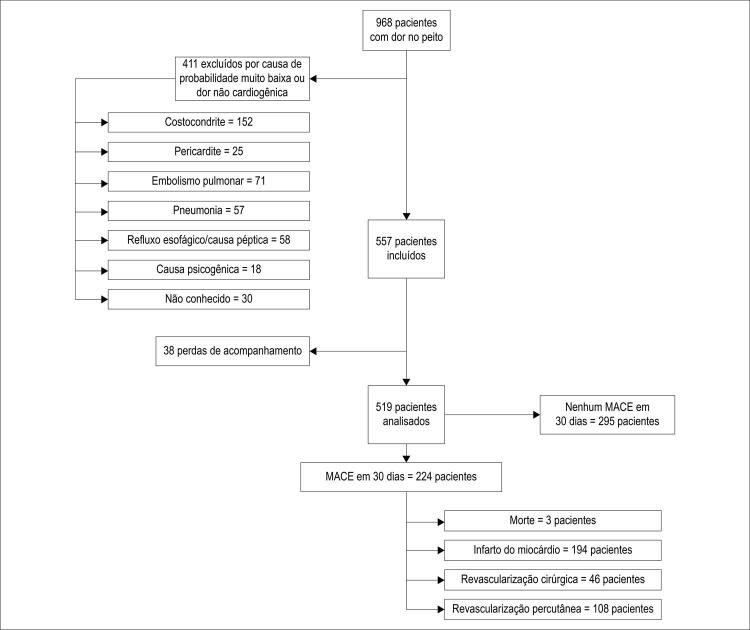
– *Pacientes do estudo.*

Os pacientes foram recrutados entre agosto de 2017 e fevereiro de 2018. O fluxo de pacientes do presente estudo está apresentado na Figura 1 .

Em total, foram incluídos 519 pacientes na análise, com um período de acompanhamento de 30 dias. As características de base dos pacientes estão apresentadas na Tabela 1 . MACE foram confirmadas em 224 pacientes durante os primeiros 30 dias de acompanhamento, com um total de 351 eventos (IAM, revascularização ou morte), correspondendo a uma incidência de MACE de 43% e uma média de 1,56 MACE por paciente com o desfecho primário. Foi diagnosticado o IMSST em 194 pacientes. Destes, 108 foram submetidos a revascularização percutânea; 46 foram submetidos a revascularização cirúrgica e 3 morreram.

**Tabela 1:** – Características da população de pacientes com e sem eventos cardiovasculares no período de 30 dias

	População (n = 519)	Com MACE (n = 224)	Sem MACE (n = 295)
Idade média (%)	64,31 (12,11%)	66,9 (11,69%)	62,3 (13,7%)
Sexo masculino, n (%)	291 (56,06%)	207 (59,5%)	84 (40,5%)
Sem fatores de risco cardiovasculares (IMC > 30, tabagismo, DM2, doença arterial coronariana familiar, idade < 55 anos, hipertensão, hipercolesterolemia)	64(12,3%)	40 (40%)	24 (60%)
1 – 2 fatores de risco	348 (67%)	247 (70,97%)	101 (29,02%)
3 ou mais fatores de risco	98 (18,8%)	74 (75,5%)	24 (24,5%)
Cardiopatia coronária prévia como o único fator de risco	84 (16,1%)	61 (72,6%)	23 (27,3%)

*DM2: diabetes mellitus tipo 2; IMC: índice de massa corporal.*

### Comparação dos escores HEART, GRACE e TIMI

A estratificação de risco para cada escore está apresentada na Tabela 2 . Com base no escore HEART, os pacientes nos grupos de risco baixo, intermediário e alto tiveram incidências de MACE de 3,1%, 46,2% e 93,7%, respectivamente. A taxa de MACE no grupo de baixo risco calculado de acordo com o escore HEART foi menor que aquelas apresentadas nos grupos de baixo risco calculados pelos outros dois escores.

**Tabela 2:** – Ocorrência de MACE (IAM, revascularização percutânea, revascularização cirúrgica ou morte) de acordo com os grupos de risco

Pontuação no HEART	Pacientes (n)	MACE (n)	MACE (%)
Baixo (0 – 3)	194	6	3,1
Intermediário (4 – 6)	182	84	46,2
Alto (7 – 10)	143	134	93,7
**Pontuação no TIMI**			
Baixo (1 – 2)	336	21	10,1
Intermediário (3 – 4)	130	119	55,6
Alto (5 – 7)	53	84	86,6
**Pontuação no GRACE**			
Baixo (< 88)	183	65	22,2
Intermediário (89 – 118)	165	88	60,7
Alto (> 118)	171	71	87,7

* MACE totais = 351. ^†^ MACE por paciente: 351 MACE / 224 pacientes = 1,56 MACE/paciente.

A pontuação ≤ 3 no escore HEART teve uma sensibilidade de 99,5% e um valor preditivo negativo (VPN) de 99% por predizer MACE na categoria de baixo risco ( Tabela 3 ). Ambos os parâmetros eram mais elevados que aqueles obtidos pelos outros escores para os grupos de baixo risco de MACE (TIMI: sensibilidade 90%, VPN 89,9%; GRACE: sensibilidade 70%, VPN 77,8%).

**Tabela 3:** – Características operativas os escores HEART, TIMI e GRACE

	HEART ≤ 3 (IC 95%)	TIMI ≤ 2 (IC 95%)	GRACE ≤ 108 (IC 95%)
SENS	99,5% (97 – 99,9)	90% (86 - 94)	70,9% (64,5 – 76,8)
ESPEC	36,6% (31,1 – 42,4)	63% (57,5 – 68,8)	77,2% (72 – 81,9)
VPN	99% (95 – 99)	89,9% (85 – 91,9)	77,8% (72,3 – 82,3)
VPP	54% (48 – 97)	65,2% (59,6 – 75,6)	70,3% (64 – 76)
RV (+)	1,57 (1,4 – 1,7)	2,47 (2,1 – 2,8)	3,125 (2,4 – 3,9)
RV (−)	0,012 (0,001 – 0,08)	0,147 (0,09 – 0,22)	0,375 (0,3 – 0,4)

* ESPEC: especificidade; RV: razão de verossimilhança; SENS: sensibilidade; VPN: valor preditivo negativo; VPP: valor preditivo positivo.

**Figura 2:**
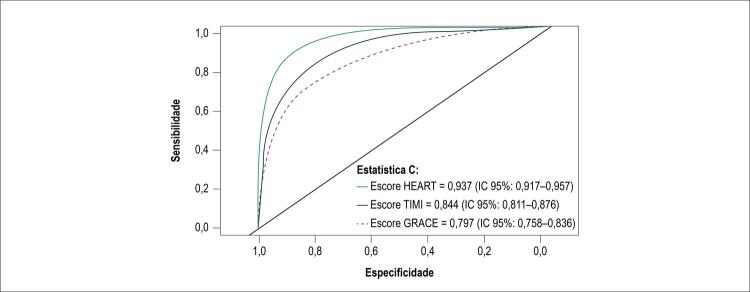
– *Curvas ROC para os escores HEART, TIMI e GRACE.*

As curvas ROC para cada escore estão apresentados na Figura 2 . A estatística C para o escore HEART foi 0,937, o qual era mais alto do que os outros dois, e foi encontrada uma diferença estatisticamente significativa utilizando o teste não paramétrico de DeLong (p < 0,0001).

**Figura 3:**
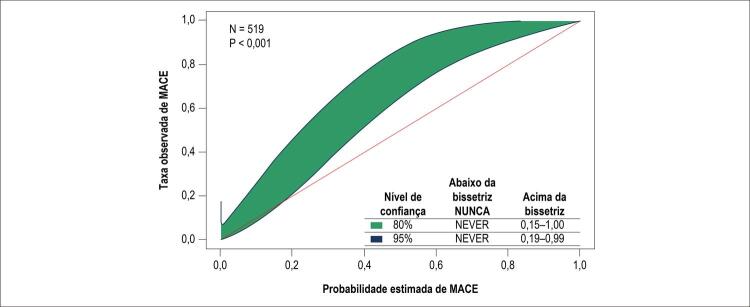
– *Teste do cinto de calibração para o escore HEART.*

Por fim, o teste do cinto de calibração do GiViTi foi utilizado para comparar resultados esperados e observados ( Figura 3 ), demonstrando calibração adequada do escore HEART para pacientes com baixo risco de MACE.^20^

## Discussão

Nós verificamos que o escore HEART para pacientes com dor no peito é uma ferramenta confiável para predizer desfechos cardiovasculares maiores, com base na descrição de sintomas do paciente, dados do registro clínico, achados eletrocardiográficos e valor inicial da TnI-as. O escore é facilmente aplicável; não requer cálculos computarizados e tem sido validado por estudos nacionais multicêntricos em múltiplas populações.^10 , 11 , 14 , 15^

Em contraste, o escore GRACE é um modelo para predizer a mortalidade em pacientes com síndrome coronariana aguda que tem sido adequadamente validado, mas o fato que deve ser calculado eletronicamente limita a sua aplicabilidade.^17^ Similarmente, o escore TIMI foi elaborado para determinar a necessidade para terapia agressiva em pacientes com síndrome coronariana aguda, permitindo o cálculo do risco por meio de variáveis dicotômicas, sem pesar as variáveis ou levar em consideração a apresentação clínica do paciente.^16^

Os resultados do presente estudo são favoráveis para o escore HEART, com uma estatística C de 0,93; este valor indica uma capacidade excelente para predizer o risco em pacientes com dor no peito, em comparação com os escores TIMI e GRACE. Isto é consistente com o que foi previamente relatado por Six et al.,^10^ Sakamoto et al.,^11^ Backus et al.,^15^ confirmando que pontuações baixas no escore HEART^24^ são muito precisas para excluir a ocorrência de MACE nos pacientes de baixo risco com acompanhamento de 30 dias.^10 , 11 , 15 , 24^

O nosso estudo obteve uma incidência de MACE de 43%, a qual é mais alta que as de 13% e 36% relatadas na literatura.^11 , 15^ Esta taxa alta de MACE pode ser devida à distinção da instituição como um centro de referência para doença cardiovascular, o qual determina um número acima da média de pacientes com riscos intermediários e altos de cardiopatia coronária. Adicionalmente, o uso exclusivo de TnI-as durante este estudo pode justificar a taxa mais alta de detecção de MACE, em comparação com aquela previamente relatada. Porém, apesar de taxas mais altas de MACE, independente do estado de risco, o escore HEART manteve a sua precisão preditiva, superando os escores TIMI e GRACE.

Em relação à sensibilidade e o VPN dos testes, nós verificamos que a estatística C foi mais alta para o escore HEART ao usar um valor de corte de 3 pontos, o qual é o limite para a categoria de baixo risco. Tanto a sensibilidade quanto o VPN são próximos a 100% e são significativamente mais elevados que a sensibilidade e o VPN dos outros dois escores. Com base nestes resultados, pode-se concluir que uma pontuação abaixo de 3 no escore HEART identifica os pacientes que podem ser tratados com uma estratégia conservadora de maneira segura, com alta precisão, visto que o risco de desfechos cardiovasculares adversos é baixo.

Adicionalmente, de acordo com o método do cinto de calibração do GiViTi, foi observado que há calibração adequada entre os desfechos esperados e observados para o grupo de baixo risco do escore HEART, em contraste com os grupos de baixo risco dos outros dois escores analisados. Isto corrobora o uso do escore HEART como um escore de primeira linha para estratificar o risco em pacientes com dor no peito com suspeita de origem cardíaca. Adicionalmente, considerando a sua facilidade de aplicação e a sua validação adequada, pode ser uma ferramenta valiosa para aprimorar a tomada de decisão e a distribuição apropriada dos recursos. Isso foi demonstrado por Mahler et al.,^12^ com o uso do “caminho HEART,” o qual combina a aplicação do escore com dosagem de troponina no momento de apresentação e 3 horas mais tarde. Este caminho resultou em uma redução significativa de testes desnecessários e uma estadia hospitalar mais breve.^12 , 25^

Até onde sabemos, nenhum outro estudo relatou o desempenho dos escores de risco na era do TnI-as em uma população exclusivamente latino-americana. A natureza prospectiva do estudo fortalece os achados. Portanto, estes resultados servem como uma validação de achados prévios em relação ao escore HEART, e devem incentivar outros projetos multicêntricos com populações maiores. Também acreditamos que estes resultados devem expandir o uso do escore HEART como uma ferramenta valiosa que visa facilitar a tomada de decisão em uma população desafiadora de pacientes.

### Limitações

Os escores TIMI e GRACE foram desenvolvidos como ferramentas para quantificar risco em pacientes com diagnóstico estabelecido de síndrome coronária aguda, enquanto o escore HEART foi elaborado para avaliar pacientes com dor no peito. Porém, apesar das diferenças nos seus objetivos iniciais, na prática clínica do mundo real, eles têm sido utilizados de forma intercambiável. Além disso, estudos prévios têm comparado os escores para avaliação de risco de dor no peito em situações de emergência.

O protocolo de pesquisa do presente estudo foi realizado em um único centro especializado, o qual pode não refletir o comportamento de outras populações em centros com níveis diferentes de complexidade ou em outras regiões ou países. Portanto, novos estudos com populações multicêntricas maiores serão necessários no futuro para aprimorar a aplicabilidade destes achados.

Embora o tamanho da amostra tenha sido menor do que inicialmente calculado, o fato de que foi obtido um número maior de MACE (n = 224) no grupo analisado de 519 pacientes possibilitou o cálculo de uma potência adequada, acima de 80%.

Adicionalmente, fatores diferentes podem influenciar a aplicabilidade do escore, considerando que os pacientes nem sempre podem fornecer o seu histórico clínico com precisão, e por este motivo os fatores de risco podem não ser adequadamente relatados. As alterações eletrocardiográficas e as elevações de troponina podem ser insignificantes nas fases iniciais de infarto do miocárdio ou podem ser falsamente elevadas por outros distúrbios, como doença renal crônica, insuficiência cardíaca, arritmias, taquicardia e sepse, entre outros.

Finalmente, a informação do acompanhamento baseia-se nos dados fornecidos pelos pacientes e seus familiares, o que pode limitar a confiabilidade dos dados. Embora as informações baseiem-se no formato estruturado com 4 perguntas claras, podem estar sujeitas a erros de interpretação.

## Conclusões

Nós verificamos que o escore HEART foi mais eficaz na previsão de MACE com 30 dias de acompanhamento em comparação com os escores TIMI e GRACE na era da TnI-as em uma população exclusivamente latino-americana com dor no peito com suspeita de origem cardíaca em um centro cardiovascular de alta complexidade.

O uso de TnI-as manteve o desempenho superior previamente demonstrado do escore HEART em comparação com os escores TIMI e GRACE.

O escore HEART possibilita a diferenciação mais precisa dos pacientes com baixo risco de apresentar MACE, o qual permitirá que os médicos optem por altas hospitalares de maneira mais precoce, possibilitando a economia de horas de estadia no hospital e testes diagnósticos desnecessários. Isto pode resultar em cuidados melhores para pacientes e na distribuição mais eficiente dos recursos dos sistemas de saúde.

## References

[1] 1. Nawar EW, Niska RW, Xu J. National Hospital Ambulatory Medical Care Survey: 2005 emergency department summary. Adv Data. 2007(386):1-32. 17703794

[2] 2. Roberts RR, Zalenski RJ, Mensah EK, Rydman RJ, Ciavarella G, Gussow L, et al. Costs of an emergency department-based accelerated diagnostic protocol vs hospitalization in patients with chest pain: a randomized controlled trial. JAMA. 1997;278(20):1670-6. 9388086

[3] 3. Kanzaria HK, Hoffman JR, Probst MA, Caloyeras JP, Berry SH, Brook RH. Emergency physician perceptions of medically unnecessary advanced diagnostic imaging. Acad Emerg Med. 2015;22(4):390-8. 10.1111/acem.12625 25807868

[4] 4. Gallardo-Solarte K, Acosta FPB, Jiménez RR. Costos de la enfermedad crónica no transmisible: la realidad colombiana. Rev Cienc Salud. 2016;14(1):103-14.

[5] 5. Observatorio Nacional de Salud. Quinto Informe ONS: carga de enfermedad por enfermedades crónicas no transmisibles y discapacidad en Colombia. Colombia: ONS; 2015.

[6] 6. Observatorio de la Seguridad Social. Grupo de Economía de la Salud GES. Evaluación económica en salud: tópicos teóricos y aplicaciones en Colombia. Universidad de Antioquia. 2006;5(14):1-16.

[7] 7. Storrow AB, Gibler WB. Chest pain centers: diagnosis of acute coronary syndromes. Ann Emerg Med. 2000;35(5):449-61. 10783407

[8] 8. Redberg RF. Getting to best care at lower cost. JAMA Intern Med. 2013;173(2):91-2. 10.1001/jamainternmed.2013.1271 22961503

[9] 9. Groarke J, O’Brien J, Go G, Susanto M, Owens P, Maree AO. Cost burden of non-specific chest pain admissions. Ir J Med Sci. 2013;182(1):57-61. 10.1007/s11845-012-0826-5 22552895

[10] 10. Six AJ, Cullen L, Backus BE, Greenslade J, Parsonage W, Aldous S, et al. The HEART score for the assessment of patients with chest pain in the emergency department: a multinational validation study. Crit Pathw Cardiol. 2013;12(3):121-6. 10.1097/HPC.0b013e31828b327e 23892941

[11] 11. Sakamoto JT, Liu N, Koh ZX, Fung NX, Heldeweg ML, Ng JC, et al. Comparing HEART, TIMI, and GRACE scores for prediction of 30-day major adverse cardiac events in high acuity chest pain patients in the emergency department. Int J Cardiol. 2016 Oct 15;221:759-64. 10.1016/j.ijcard.2016.07.147 27428317

[12] 12. Mahler SA, Riley RF, Hiestand BC, Russell GB, Hoekstra JW, Lefebvre CW, et al. The HEART Pathway randomized trial: identifying emergency department patients with acute chest pain for early discharge. Circ Cardiovasc Qual Outcomes. 2015;8(2):195-203. 10.1161/CIRCOUTCOMES.114.001384 PMC4413911 25737484

[13] 13. Fanaroff AC, Rymer JA, Goldstein SA, Simel DL, Newby LK. Does this patient with chest pain have acute coronary syndrome?: the rational clinical examination systematic review. JAMA. 2015;314(18):1955-65. 10.1001/jama.2015.12735 26547467

[14] 14. Six AJ, Backus BE, Kelder JC. Chest pain in the emergency room: value of the HEART score. Neth Heart J. 2008;16(6):191-6. 10.1007/BF03086144 PMC2442661 18665203

[15] 15. Backus BE, Six AJ, Kelder JC, Bosschaert MA, Mast EG, Mosterd A, et al. A prospective validation of the HEART score for chest pain patients at the emergency department. Int J Cardiol. 2013;168(3):2153-8. 10.1016/j.ijcard.2013.01.255 23465250

[16] 16. Antman EM, Cohen M, Bernink PJ, McCabe CH, Horacek T, Papuchis G, et al. The TIMI risk score for unstable angina/non-ST elevation MI: A method for prognostication and therapeutic decision making. JAMA. 2000;284(7):835-42. 10.1001/jama.284.7.835 10938172

[17] 17. Eagle KA, Lim MJ, Dabbous OH, Pieper KS, Goldberg RJ, Van de Werf F, et al. A validated prediction model for all forms of acute coronary syndrome: estimating the risk of 6-month postdischarge death in an international registry. JAMA. 2004;291(22):2727-33. 10.1001/jama.291.22.2727 15187054

[18] 18. Wagner GS, Macfarlane P, Wellens H, Josephson M, Gorgels A, Mirvis DM, et al. AHA/ACCF/HRS recommendations for the standardization and interpretation of the electrocardiogram: part VI: acute ischemia/infarction: a scientific statement from the American Heart Association Electrocardiography and Arrhythmias Committee, Council on Clinical Cardiology; the American College of Cardiology Foundation; and the Heart Rhythm Society. Endorsed by the International Society for Computerized Electrocardiology. J Am Coll Cardiol. 2009;53(11):1003-11. 10.1016/j.jacc.2008.12.016 19281933

[19] 19. Taylor J. Third universal definition of myocardial infarction. Eur Heart J. 2012;33(20):2506-7. 10.1093/eurheartj/ehs296 23065972

[20] 20. Ibánez B, James S, Agewall S, Antunes MJ, Bucciarelli-Ducci C, Bueno H, et al. 2017 ESC Guidelines for the management of acute myocardial infarction in patients presenting with ST-segment elevation. Rev Esp Cardiol (Engl Ed). 2017;70(12):1082. 10.1016/j.rec.2017.11.010 29198432

[21] 21. Simel DL, Samsa GP, Matchar DB. Likelihood ratios with confidence: sample size estimation for diagnostic test studies. J Clin Epidemiol. 1991;44(8):763-70. 10.1016/0895-4356(91)90128-v 1941027

[22] 22. Youden WJ. Index for rating diagnostic tests. Cancer. 1950;3(1):32-5. 10.1002/1097-0142(1950)3:1<32::aid-cncr2820030106>3.0.co;2-3 15405679

[23] 23. Finazzi S, Poole D, Luciani D, Cogo PE, Bertolini G. Calibration belt for quality-of-care assessment based on dichotomous outcomes. PLoS One. 2011;6(2):e16110. 10.1371/journal.pone.0016110 PMC3043050 21373178

[24] 24. Stopyra JP, Harper WS, Higgins TJ, Prokesova JV, Winslow JE, Nelson RD, et al. Prehospital Modified HEART score predictive of 30-day adverse cardiac events. Prehosp Disaster Med. 2018;33(1):58-62. 10.1017/S1049023X17007154 29316995

[25] 25. Hyams JM, Streitz MJ, Oliver JJ, Wood RM, Maksimenko YM, Long B, et al. Impact of the HEART pathway on admission rates for emergency department patients with chest pain: an external clinical validation study. J Emerg Med. 2018;54(4):549-57. 10.1016/j.jemermed.2017.12.038 29478861

