# Supplementary material for: Os Escores HEART, TIMI e GRACE para Predição de Eventos Cardiovasculares Adversos Maiores no Período de 30 Dias na Era de Troponina I de Alta Sensibilidade
Source: Arq Bras Cardiol. 2020 May 22;114(5):795–802. [Article in Portuguese] doi: 10.36660/abc.20190206 (PMC8387004; doi:10.36660/abc.20190206)
Supplement: Translation

# Translation: HEART, TIMI, and GRACE Scores for Prediction of 30-Day Major Adverse Cardiovascular Events in the Era of High-Sensitivity Troponin

**Authors:** Felipe Torralba, Alberto Navarro, Juan Castellanos-de la Hoz, Carlos Ortiz, Alberth Botero, Freddy Alarcón, Nicolas Isaza, Daniel Isaza

**Affiliations:** 1 Colombia Fundacion Cardioinfantil Instituto de Cardiologia, Bogota Cundinamarca – Colombia

**Keywords:** Cardiovascular Diseases/mortality, Chest Pain, Myocardial Infarction, Forecasting Risk Assessment, Risk Factors, Troponin, Myocardial Ischemia

## Abstract

**Background:**

Multiple scoring systems have been designed to calculate the risk of major adverse cardiovascular events (MACE) in patients with chest pain. There is no data on whether the HEART score outperforms TIMI and GRACE in the prediction of MACE, especially in the era of high-sensitivity troponin assay and in an exclusively Latin-American population.

**Objective:**

To compare the performance of the HEART, TIMI, and GRACE scores for predicting major cardiovascular events at 30 days of follow-up, in patients who consult for chest pain in the emergency department.

**Methods:**

HEART, TIMI, and GRACE scores were analyzed in 519 patients with chest pain at the emergency department. The primary endpoint was the occurrence of MACE within 30 days. The performance of the HEART score was compared with the TIMI and GRACE scores using the DeLong test with p values of 0.05 considered statistically significant.

**Results:**

A total of 224 patients (43%) had MACE at 30 days. The C statistic for the HEART, TIMI, and GRACE score was 0.937, 0.844, and 0.797 respectively (p < 0.0001). A HEART score of 3 or less had a sensitivity of 99.5% and a negative predictive value of 99% to classify low risk patients correctly; both values were higher than those obtained by the other scores.

**Conclusion:**

The HEART score more effectively predicts cardiovascular events at 30 days of follow-up compared to the other scores. High-sensitivity troponins maintain this score’s previously demonstrated superiority. This score offers more precise identification of low-risk patients. (Arq Bras Cardiol. 2020; [online].ahead print, PP.0-0)

## Introduction

Chest pain is one of the most common complaints in patients presenting to the emergency department, with approximately 15 million patient visits in the United States and Europe.^1^ It is estimated that 55% of these patients have a non-cardiac cause for chest pain and only one fifth are definitively diagnosed with acute coronary syndromes.^1 , 2^ Approximately 85% of patients with chest pain are admitted, in spite of the fact that up to 60% of cases could be managed in the outpatient setting.^3^

In Colombia, cardiovascular diseases are also a cause of high mortality; among these, ischemic heart disease was the main cause in the previous decade, accounting for 49.5% of the total in this group.^4 , 5^ The annual cost of treatment for patients with chest pain of non-cardiac cause can be as high as 8 billion dollars in the USA and approximately 3.9 billion dollars in Colombia.^6^ These expenses originate primarily from daily bed costs and radiological and laboratory studies.^2 , 7 , 8^ This significant economic impact has driven efforts to develop alternatives that enable more efficient use of resources, particularly in countries with limited health budgets.^3 , 8 , 9^

The development of a tool to accurately determine the risk of major adverse cardiovascular events (MACE) in these patients is essential, and scoring systems such as TIMI and GRACE have been designed to address this problem.^10 , 11^ More recently, the HEART score was created, being the first one prospectively designed to predict MACE.^12 - 14^

The HEART score has outperformed the TIMI and GRACE scores in Asian, European, and North American populations.^11 , 15^ This study aimed to compare the accuracy of these scores for predicting MACE in a group of Latin-American patients with chest pain who presented to a cardiovascular reference center. To the best of our knowledge, this is the first prospective study of this nature.

## Methods

This is a prospective observational study of diagnostic tests carried out in the Fundación Cardioinfantil, located in Bogotá, Colombia. It is a high-complexity hospital specialized in cardiovascular medicine, with a monthly average of 9,000 emergency consultations, 15% of which correspond to cardiovascular emergencies.

Patients over 18 years of age who presented at the emergency department with acute chest pain between August 2017 and February 2018 were included in the study. According to the institutional protocol, patients were evaluated by the cardiologist; electrocardiography was performed, and high sensitivity troponin I (hsTnI) was measured, initially and 3 hours later if needed, using ARCHITECT STAT assay (Abbot, Lake Bluff, IL, USA).

Acute myocardial infarction (AMI) was diagnosed when hsTnI values were greater than 0.026 ng/mL (reference value 0.0 – 0.026 ng/ml). In this case, patients were admitted for in-hospital care, coronary arteriography, and either percutaneous or surgical revascularization. When values were negative, but pain was considered of intermediate or high probability, the patients were admitted for further evaluation with a non-invasive stratification strategy.

Patients with myocardial infarction with ST elevation and non-cardiac causes of chest pain, such as pneumonia, trauma, or psychogenic pain, were excluded from this study.

The HEART, TIMI, and GRACE risk scores were calculated for this group of patients at the time of consultation.

### Risk Scores

The methods for calculating the GRACE, TIMI, and HEART scores have been described in previous articles and are briefly summarized below.^14 , 16 , 17^ The score calculations were performed using the information documented in the electronic medical record, the first electrocardiogram upon presentation, and the first laboratory values measured, including troponin measurement with the hsTnI assay.

The HEART score consists of the following 5 categorical variables: the patient’s medical history, electrocardiogram, age, risk factors for coronary heart disease, and troponin. Each variable has a maximum value of 2 points adding up to a maximum score of 10, which indicates a patient with maximum risk. The GRACE score consists of the following 5 categorical variables: age, heart rate, blood pressure, creatinine, and Killip class; and the following 3 nominal variables: cardiac arrest, ST-segment deviation, and troponin elevation. Each item is assigned a value, and the sum of these values determines the risk of MACE. Finally, the TIMI score consists of the following 7 dichotomous nominal variables: age over 65 years, more than 3 risk factors for coronary artery disease, significant coronary artery stenosis, symptoms of severe angina, ST-segment deviation, use of aspirin in the last week, and elevation of troponin. The maximum TIMI score is 7, with higher scores indicating higher risks.

### Ethics

The study was carried out in accordance with the principles laid out in the Declaration of Helsinki of the World Medical Association, the Nuremberg Code, and the World Health Organization International Ethical Guidelines for research involving humans, as well as domestic regulations related to basic health care. The study received approval from the ethics and research committee of the Fundación Cardioinfantil, and the patients included in the study provided their informed consent.

### Data management

The data included demographic information of the patients, as well as information on clinical history, laboratory values, electrocardiographic findings, and vital signs.

Laboratory values included creatinine and hsTnI, which are the assays currently applied in the institutional protocol.

The attending cardiologist evaluated the 12-lead electrocardiogram according to the guidelines of the American Heart Association.^18^ If necessary, the exam was submitted to a second blinded attending cardiologist for evaluation.

An encrypted database was created to which only the authors of the study had access, and an algorithm was developed for the automatic calculation of the risk scores.

### Follow-up

Follow-up was performed at 30 days, reviewing the electronic medical record and employing a telephone survey. A structured format was applied with 4 clear questions regarding the occurrence of major cardiovascular events (death, myocardial infarction, surgical revascularization, or percutaneous revascularization), to determine the presence of the primary outcome.

### Outcomes

The diagnosis of AMI was made when troponin values rose above the 99th percentile of reference values (hsTnI > 0.026 ng/mL), and evidence of myocardial ischemia was documented on electrocardiogram. We applied the criteria described by the third universal definition of AMI, which was valid at the time the protocol was written.^19^ We also defined ST-elevation myocardial infarction (STEMI), non-ST-elevation myocardial infarction (NSTEMI), and signs of ischemia according to the guidelines validated at the time of protocol design.^20^

Percutaneous revascularization was defined as any intervention through a catheter in the coronary arteries, and surgical revascularization was defined as any cardiac surgery in which coronary artery grafts were made. MACE were defined as death from any cause, myocardial infarction, and surgical or percutaneous myocardial revascularization. Follow up was completed 30 days after admission to the emergency department.

### Statistical analysis

We calculated a sample of 550 patients, to obtain 185 MACE using Simel and Samsa’s method of maximum sensitivity,^21^ in order to yield a power of 80% and a confidence interval of 95% with an alpha error of 5%. For each score, the best cutoff point was calculated using the Youden index,^22^ considering p values of 0.05 significant. Subsequently, the C statistic, the positive and negative likelihood ratio (LR), sensitivity, and specificity were calculated. Then, the LR was calculated for each risk stratum. The difference between LR was calculated using the test for adequate binomial proportions (Chi-square test and Fisher’s exact test), considering p values of 0.05 significant.

The area under the curve for each test was calculated and compared using the nonparametric DeLong test (p = 0.05), and, finally, a calibration test was also made for each score to compare expected and actual major cardiovascular events in the study population, according to the calibration belt method described by Finazzi S, et al.^23^ from the Italian Group for the Evaluation of Interventions in Intensive Care Medicine (GiViTi).^23^

Analysis was carried out using the statistical program R, version 3.3.3 (the R Foundation for Statistical Computing, Vienna, Austria).

## Results

Patients were recruited between August 2017 and February 2018. The present study’s patient flow is shown in Figure 1 .

**Figure 1:** – *Patients of the study.*

A total of 519 patients were included in the analysis, with a follow-up period of 30 days. Baseline patient characteristics are shown in Table 1 . MACE were confirmed in 224 patients within the first 30 days of follow-up, with a total of 351 events (AMI, revascularization, or death). These account for a MACE incidence of 43% and an average of 1.56 MACE per patient with the primary outcome. NSTEMI was diagnosed in 194 patients. Of these patients, 108 underwent percutaneous revascularization; 46 underwent surgical revascularization, and 3 died.

**Table 1:** – Population characteristics of patients with and without cardiovascular events at 30 days

	Population (n = 519)	MACE (n = 224)	No MACE (n = 295)
Average age (%)	64.31 (12.11%)	66.9 (11.69%)	62.3 (13.7%)
Male sex, n (%)	291 (56.06%)	207 (59.5%)	84 (40.5%)
Without cardiovascular risk factors (BMI > 30, smoker, DM2, family coronary artery disease, age < 55 years, hypertension, hypercholesterolemia)	64 (12.3%)	40 (40%)	24 (60%)
1 – 2 risk factors	348 (67%)	247 (70.97%)	101 (29.02%)
3 or more risk factors	98 (18.8%)	74 (75.5%)	24 (24.5%)
Previous coronary heart disease as the only factor	84 (16.1%)	61 (72.6%)	23 (27.3%)

*BMI: body mass index; DM2: diabetes mellitus type 2.*

### HEART, GRACE, and TIMI score comparison

Risk stratification for each score is shown in Table 2 . Based on the HEART score, patients in the low, intermediate, and high risk groups had 3.1%, 46.2%, and 93.7% incidence of MACE, respectively. The MACE rate in the low-risk group calculated according to the HEART score was lower than that of the low-risk groups calculated by the other two scores.

**Table 2:** – Occurrence of MACE (AMI, percutaneous revascularization, surgical revascularization, or death) according to risk groups

HEART score	Patients (n)	MACE (n)	MACE (%)
Low (0 - 3)	194	6	3.1
Intermediate (4 – 6)	182	84	46.2
High (7 – 10)	143	134	93.7
**TIMI score**			
Low (1 – 2)	336	21	10.1
Intermediate (3 – 4)	130	119	55.6
High (5 – 7)	53	84	86.6
**GRACE score**			
Low (< 88)	183	65	22.2
Intermediate (89 – 118)	165	88	60.7
High (> 118)	171	71	87.7

* Total MACE = 351.^†^MACE per patient: 351 MACE / 224 patients = 1.56 MACE / patient.

A HEART score ≤ 3 had a sensitivity of 99.5% and a negative predictive value (NPV) of 99% to predict MACE in the low risk category ( Table 3 ). Both parameters were higher than those obtained with the other scores for the low risk MACE group (TIMI: sensitivity 90%, NPV 89.9%; GRACE: sensitivity 70%, NPV 77.8%).

**Table 3:** – Operative characteristics for the HEART, TIMI, and GRACE scores

	HEART score ≤ 3 (CI 95%)	TIMI score ≤ 2 (CI 95%)	GRACE score ≤ 108 (CI 95%)
SENS	99.5% (97 – 99.9)	90% (86 - 94)	70.9% (64.5 – 76.8)
SPEC	36.6% (31.1 – 42.4)	63% (57.5 – 68.8)	77.2% (72 – 81.9)
NPV	99% (95 - 99)	89.9% (85 – 91.9)	77.8% (72.3 – 82.3)
PPV	54% (48 - 97)	65.2% (59.6 – 75.6)	70.3% (64 - 76)
LR (+)	1.57 (1.4 – 1.7)	2.47 (2.1 – 2.8)	3.125 (2.4 – 3.9)
LR (−)	0.012 (0.001 – 0.08)	0.147 (0.09 – 0.22)	0.375 (0.3 – 0.4)

* SENS: sensitivity; SPEC: specificity; NPV: negative predictive value; PPV: positive predictive value; LR: likelihood ratio.

The ROC curves for each score are shown in Figure 2 . The C statistic for the HEART score was 0.937, which was higher than the other two scores, and a statistically significant difference was found using the nonparametric DeLong test (p < 0.0001).

**Figure 2:** – *ROC curves for the HEART, TIMI and GRACE scores.*

Finally, the GiViTi calibration belt test was used to compare expected and observed results ( Figure 3 ), showing adequate calibration of the HEART score for patients with low MACE risk.^20^

**Figure 3:** – *Calibration belt test for HEART score*

## Discussion

We found that the HEART score for patients with chest pain is a reliable tool for predicting major cardiovascular outcomes based on the patients’ description of symptoms, clinical record data, electrocardiographic findings, and initial hsTnI value. It is readily applicable; it does not require computerized calculations, and it has been validated by international multicenter studies in multiple populations.^10 , 11 , 14 , 15^

Conversely, the GRACE score is a model for predicting mortality in patients with acute coronary syndrome that has been adequately validated, but the fact that it must be calculated electronically limits its applicability.^17^ Similarly, the TIMI score was designed to determine the need for aggressive therapy in patients with acute coronary syndrome, allowing the calculation of risk through the use of dichotomous variables without weighing the variables or taking patient’s clinical presentation into account.^16^

The results of this study are favorable for the HEART score, with a C statistic value of 0.93, which indicates an excellent ability to predict the risk of patients with chest pain, compared to the TIMI and GRACE scores. This is consistent with what was previously reported by Six et al.,^10^ Sakamoto et al.,^11^ Backus et al.,^15^ and confirming that low scores on the HEART^24^ scale are very accurate for ruling out the occurrence of MACE in low-risk patients with a 30-day follow up.^10 , 11 , 15 , 24^

Our study had a MACE incidence of 43%, which is higher than the 13% and 36% reported in the literature.^11 , 15^ This high rate of MACE might be due to the institution’s distinction as a referral center for cardiovascular disease, which leads to a higher than average number of patients with intermediate and high risks of coronary heart disease. Additionally, the exclusive use of hsTnI during this study might explain a higher rate of MACE detection than previously reported. However, despite higher rates of MACE, irrespective of risk status, the HEART score maintained its predictive precision, outperforming both the TIMI and GRACE scores.

With regards to the sensitivity and the NPV of the tests, we found that the C statistic was higher for the HEART score when using a cutoff of 3 points, which is the limit for the low-risk category. Both the sensitivity and NPV are close to 100%, and they are significantly higher than the sensitivity and NPV of the other two scores. Based on these results it can be concluded that a HEART score below 3 identifies patients that can safely be managed with a conservative strategy with high certainty, given that the risk of adverse cardiovascular outcomes is low.

Additionally, according to the GiViTi belt method, it is observed that there is adequate calibration between expected and observed outcomes for the low-risk group in the HEART score, as opposed to the low-risk groups in the two other scores analyzed. This supports the potential use of the HEART score as a first line score to stratify risk in patients with chest pain of suspected cardiac origin. Additionally, given its ease of application and adequate validation, it can be a valuable tool to enhance decision making and proper distribution of resources. This has been demonstrated by Mahler et al.,^12^ with the use of the “HEART pathway,” which combines the application of this score with troponin testing upon presentation and 3 hours later. This pathway led to a significant reduction of unnecessary tests and a shorter total hospital stay.^12 , 25^

To the best of our knowledge, no other studies have reported the performance of risk scores conducted in the era of hsTnI in an exclusively Latin-American population. The prospective nature of the study strengthens the findings. Therefore, these results serve as a validation of previous findings regarding the HEART score, and they should motivate further multicentric projects with larger populations. Also, we believe that these results should expand the use of the HEART score as a valuable tool that aims to facilitate decision making in a challenging patient population.

### Limitations

The TIMI and GRACE score were developed as tools to quantify risk in patients with an established diagnosis of acute coronary syndrome, whereas the HEART score was designed to assess patients with chest pain. However, despite the difference in their initial objective, in real-world clinical practice they have been used interchangeably. Furthermore, previous studies have compared the scores for risk assessment of chest pain in emergency settings.

This research protocol was carried out in a single specialized center, which may not accurately reflect the behavior of other populations in centers with different levels of complexity or in different regions or countries. Therefore, new studies with larger, multicentric populations will be required in the future to enhance the applicability of these findings.

Although the sample size was smaller than initially calculated, the fact that a greater number of MACE (n = 224) was obtained in the analyzed group of 519 patients made it possible to calculate an adequate power greater than 80%.

Additionally, different factors may affect score applicability, as patients may not always provide accurate clinical history, and therefore risk factors may not be adequately reported. Electrocardiographic changes and troponin elevations may be non-significant in the early stages of myocardial infarction, or they may be falsely elevated by other disorders such as chronic kidney disease, heart failure, arrhythmias, tachycardia, and sepsis, among others.

Finally, the follow-up information is based on the data provided by patients and their family members, which could limit the reliability of the data. Although the information is based on a structured format with 4 clear questions, it may be subject to misinterpretation.

## Conclusions

We found that the HEART score was more effective in predicting MACE at 30 days of follow up compared to the TIMI and GRACE scores in the era of hsTnI in an exclusively Latin-American population with chest pain of suspected cardiac origin at a high complexity cardiovascular center.

The use of hsTnI maintained the previously demonstrated superior performance of the HEART score compared to the TIMI and GRACE scores.

The HEART score allows for more accurate differentiation of patients with low risks of presenting major cardiovascular events, which will enable physicians to opt for earlier discharge and which may allow savings in hours of in-hospital stay and unnecessary diagnostic tests. This could lead to better care for patients and more efficient distribution of healthcare system resources.
